# Cybersecurity in PACS and Medical Imaging: an Overview

**DOI:** 10.1007/s10278-020-00393-3

**Published:** 2020-10-29

**Authors:** Marco Eichelberg, Klaus Kleber, Marc Kämmerer

**Affiliations:** 1 R&D Department Health, OFFIS-Institute for Information Technology, Escherweg 2, 26121 Oldenburg, Germany; 2 VISUS Health IT GmbH, Gesundheitscampus-Süd 15-17, 44801 Bochum, Germany

**Keywords:** Cybersecurity, PACS, DICOM, Medical imaging

## Abstract

This article provides an overview on the literature published on the topic of cybersecurity for PACS (Picture Archiving and Communications Systems) and medical imaging. From a practical perspective, PACS specific security measures must be implemented together with the measures applicable to the IT infrastructure as a whole, in order to prevent incidents such as PACS systems exposed to access from the Internet. Therefore, the article first offers an overview of the physical, technical and organizational mitigation measures that are proposed in literature on cybersecurity in healthcare information technology in general, followed by an overview on publications discussing specific cybersecurity topics that apply to PACS and medical imaging and present the “building blocks” for a secure PACS environment available in the literature. These include image de-identification, transport security, the selective encryption of the DICOM (Digital Imaging and Communications in Medicine) header, encrypted DICOM files, digital signatures and watermarking techniques. The article concludes with a discussion of gaps in the body of published literature and a summary.

## Introduction

The use of information technology (IT) permeates modern medicine. Starting with the introduction of hospital information systems (HIS) around 1970, digital imaging modalities such as computed tomography (CT) and magnetic resonance imaging (MRI) in the 1970s and 1980s, Picture Archiving and Communication Systems (PACS) and softcopy reading in the 1980s and 1990s, to the electronic sharing of clinical information across regions, nations, or even internationally today. The Internet has become an indispensable source of information and a means of communicating quickly, efficiently, and inexpensively. However, the widespread use of IT and the Internet has also created new challenges, and one topic that has become increasingly important for hospitals is *cybersecurity*, a term that the Oxford English Dictionary defines as “*the state of being protected against the criminal or unauthorized use of electronic data, or the measures taken to achieve this*.”

The concepts of “malware” (malicious software) and “hacking” (unauthorized intrusion into a computer or a network) predate the widespread adoption of the Internet and go back at least to the early 1970s. However, the fact that today most IT systems worldwide are connected to the Internet to some degree has caused a dramatic increase in such incidents, which are no longer primarily attributed to hobbyists driven by curiosity, but to organized crime and Advanced Persistent Threat (APT) groups associated with nation states [1].

A particularly harmful type of malware that currently plagues organizations and computer users worldwide is “ransomware.” This is a software that, once downloaded and started, encrypts as many files as possible and then demands the payment of a ransom (typically using a cryptocurrency such as Bitcoin), with the promise that after the payment, the decryption key for the encrypted files will be made available by the malware authors, which may or may not be the case. A document published by the US Department of Justice [2] states that 4000 ransomware attacks per day were reported in 2016, an increase by a factor of four compared to 2015. Furthermore, healthcare is among the most affected sectors, with 15% of all ransomware globally detected in a healthcare institution in 2017 [3]. Fifty percent of all cybersecurity incidents in hospitals in 2017 were related to ransomware [3].

Policy makers around the globe have recognized that healthcare institutions are part of a society’s critical infrastructure that requires protection, including protection from cyber threats. For example, the US National Infrastructure Protection Plan provides a Healthcare and Public Health Sector-Specific Plan [4] and the European Union has established the EU Agency for Network and Information Security (ENISA), which among other topics publishes studies related to cybersecurity issues in the healthcare sector [5, 6]. In Germany, large hospitals (hospitals with 30,000 or more inpatient admissions per year) had to implement the requirements of the IT Security Act, aimed at critical infrastructure providers, by July 2019.

On the other hand, the cybersecurity threats faced by hospitals have reached a new quality. While in the past attacks were often widespread and random, they have now become increasingly targeted at the healthcare sector, which is apparently seen as an attractive target by the groups behind these threats. For example, instead of generic “phishing” mail widely distributed as unsolicited e-mail (“SPAM”), asking users to open an e-mail attachment, attackers increasingly use the social engineering technique of “spear phishing” [7]. A user, e.g., an employee of the human resource department, will receive a convincingly looking e-mail with a resume document attached or linked to, which, when opened, turns out to be malware infecting the computer.

The aim of this article is to provide an overview on the literature published on the topic of cybersecurity for PACS and medical imaging, both for the researcher studying the topic and the practitioner such as a hospital’s CIO or PACS administrator who wants to compare their own cybersecurity plans with the state of the art. From a practical perspective, PACS-specific security measures must be implemented together with the measures applicable to the IT infrastructure as a whole, in order to prevent incidents such as image archives configured to permit unrestricted access over the Internet due to a lack of rather basic IT security measures. Studies by Stites et al. [8] in 2015, by Gillum et al. [9] and Greenbone Networks [10] in 2019 and a follow-up report by Whittaker [11] in 2020 show that this is a very real problem. In each case, hundreds of PACS systems around the globe were found to be exposed to access from the Internet, with numbers increasing over time.

The article is, therefore, structured as follows: the “Cybersecurity in Healthcare IT” section provides an overview of literature on cybersecurity in healthcare information technology in general. The “Cybersecurity in PACS and Medical Imaging” section provides an overview on publications discussing specific cybersecurity topics that apply to PACS and medical imaging and present the “building blocks” for a secure PACS environment available in the literature. In the “Discussion” section, we discuss the results of the review and point out gaps in the body of published literature. Finally, the “Summary” section offers a summary.

The authors have deliberately chosen not to structure this article according to the PRISMA guidelines (“Preferred Reporting Items for Systematic Reviews and Meta-Analyses”) because (a) for many of the individual topics covered in this article, reviews have been published and we prefer to direct the readers to these reviews instead of repeating their content; (b) in the field of cybersecurity, many relevant publications are standards and technical guidelines rather than rigorous scientific studies lending themselves to a PRISMA meta-analysis; and (c) the aim of summarizing the most important topics of this large field in a tutorial style that is of relevance to the practitioner.

## Review

### Cybersecurity in Healthcare IT

The field of cybersecurity has seen a dramatic increase of publications since 2012 [12]. Numerous standards, governmental regulations, best practice guidelines, and scientific papers discuss cybersecurity and provide recommendations. It is, therefore, beyond the scope of this article to provide a comprehensive overview of IT security in general. We nevertheless try to summarize the most important recommendations from a practitioner’s perspective that are applicable to healthcare IT, and to PACS and medical imaging networks in particular. For further reference, readers should consult the most important standards in this field, ISO/IEC 27002:2013 [13] and ISO 27799:2016 [14]. Furthermore, several reviews of the literature in this field have been published [12, 15–17]. A study published by the European Union Agency for Network and Information Security (ENISA) [5] provides an overview of the systems and devices in healthcare institutions and the types of threats that need to be considered in the field of cybersecurity and analyses their criticality, with the two most critical system categories being interconnected clinical information systems and networked medical devices. A whitepaper by the National Electrical Manufacturers Association (NEMA) [18] provides a list of resources that support healthcare institutions in establishing an effective IT security program. Finally, it should be noted that while many mitigation measures can be implemented by the users of a system, others must be considered as part of the system design and can only be implemented by the system developers. The following discussion will focus on measures that can be implemented by healthcare institutions. Cybersecurity requirements for system developers are discussed in detail in [19–21].

#### Physical Mitigation Measures

The first and arguably most obvious level of cybersecurity is physical: technical mitigation measures such as passwords, virus scanners or fine-grained user privileges are of little value if an attacker can simply walk into a server room and steal computers or storage media. An ENISA study [6] reports that physical and environmental security is the second top security requirement in eHealth, after incident reporting. “A basic principle for the physical protection of data is to ensure that file servers are located in secure areas safeguarded from unauthorized access and environmental threats such as fire, flood, loss of power etc.” Liu et al. [22] report that most data breaches of protected health information in the USA “occurred via electronic media, frequently involving laptop computers or portable electronic devices. Most breaches also occurred via theft.” This highlights that mobile devices, which cannot be kept behind locked doors in server rooms, are a topic that also needs to be taken into account; we will discuss this further in the next section.

A guideline by the US Department of Health and Human Services [23] explains that, “should a data storage device disappear, no matter how well an office has taken care of its passwords, access control, and file permissions, it is still possible that a determined individual could access the information on it. Therefore, it is important to limit the possibility of devices disappearing or being tampered with.” The guideline recommends that “securing devices and information physically should include policies limiting physical access, for example, securing machines in locked rooms, managing physical keys, and restricting the ability to remove devices from a secure area.” Finally, Wikina [24] mentions the installation of security cameras as a possible security measure.

#### Technical Mitigation Measures

The second level on which cybersecurity must be implemented is the level of technical mitigation measures. The majority of recommendations in literature concern this level. In this section, we present a summary of the technical mitigation measures that apply to PACS and medical imaging devices. Many of these will seem obvious, but will still require effort for an effective implementation.

- ** Regular backups:** ENISA [5] recommends the performance of regular backups. “This very important action can solve many attacks that could cause great impacts to smart hospitals such as ransomware or physical attacks. Running regular full or incremental backups can be done combined with setting a hot or warm site, making the hospital systems resilient even in the case of natural disaster.” The US Department of Justice [2] adds that operators should “ensure backups are not connected permanently to the computers and networks they are backing up. Examples are securing backups in the cloud or physically storing backups offline.” NTT Security [3] points out that a comprehensive backup strategy includes “storage of offline backups, as well as confirming the organization’s ability to rebuild systems and restore data”. Sittig et al. [25] recommend that backups “should be made frequently (i.e., at least daily, and a continuous or real-time backup is ideal).” They also recommend hospitals to “periodically conduct mock system recovery exercises (i.e., identify backups and test restore capabilities).”
- ** Firewalls and network segmentation:** Kruse et al. [26] performed an analysis of literature on security techniques for electronic health records and conclude that “the security technique most commonly discussed was the implementation of firewalls to protect the healthcare organizations’ information technology system.” They also point out that firewalls “have proven to be very successful in securing an organization’s network and the protected health information that resides on the network.” ENISA [5] points out that “it is important to separate critical parts of the network from non-critical parts. For instance, it is recommended to separate medical devices to the largest possible extent from office components that are typically—due to the use of standard components—susceptible to a wide range of attacks. Moreover, devices with known vulnerabilities that cannot be removed easily may only be used in a separate part of the network or not connected to the network at all.” NTT explains that network segmentation is important because “if attackers can breach back-end servers, they may be able to move laterally to access other portions of your network, doing further damage, and possibly gaining a foothold across multiple systems” [27]. They recommend the use of “firewalls, routers, and other network security devices to implement and enforce network segregation,” i.e., “restricting the flow of network traffic between network segments with different security profiles” [3]. They also recommend the use of “web and application gateway firewalls to help protect key internal and external applications” [27]. Recent trends in this field are micro-segmentation and the zero trust paradigm. OTech explains that “micro-segmentation allows for networks to be configured using software such that certain devices only talk with each other. If a device or application moves, the security policies and attributes move with it. Zero trust means that it is not sufficient to only protect the perimeter; nothing can be trusted anymore as devices might become infected as well, so it shifts the focus to internal protection.” [28] An introduction to micro-segmentation is provided by De Vincentis [29], and an implementation of zero trust networks is described by Vanickis et al. [30]. Finally, the use of “data diodes” has been proposed for healthcare networks. These are hardware devices that enforce a strictly unidirectional communication from one network with a high security level to another network with a lower security level. El Hajal et al. [31] describe their use in the context of a PACS network, but point out that data diodes cannot be used with the existing DICOM network protocol, which relies on bidirectional communication.
- ** Disabling unused physical ports:** One important route for the delivery of malware and for data theft is via portable storage media such as universal serial bus (USB) memory sticks. Sittig et al. [25] recommend, therefore, “that at the local device level, organizations should consider disabling USB ports to prevent malicious software delivery.” ENISA [20] recommends operators to “ensure that devices only feature the essential physical external ports (such as USB) necessary for them to function and that the test/debug modes are secure, so they cannot be used to maliciously access the devices.” In general, operators should “lock down physical ports to only trusted connections.”
- ** Whitelisting of permitted applications:** Many operating systems support the concept of “whitelisting” applications. When enabled, only applications that are included in the “whitelist” managed by the operating system can be executed, while all others are blocked. NEMA [18] reports that “whitelisting mechanisms can effectively preclude execution of malware code and can be integrated into a device prior to commissioning.” NTT [3] recommends that organizations should, “if feasible, use application whitelisting on servers, desktops, and laptops so ransomware and other unauthorized executables can’t be run.” This requires organizations to develop “a ‘whitelist’ of specified programs that are allowed to run.” [25], which should be relatively simple in the PACS context where only a limited number of applications will be used, e.g., on a diagnostic workstation, but might be a rather complex task on general-purpose office PCs. As an alternative where whitelisting is not possible, the US guidance document on ransomware protection recommends users [2] to “implement Software Restriction Policies (SRP) or other controls to prevent programs from executing from common ransomware locations, such as temporary folders supporting popular Internet browsers or compression/decompression programs, including the AppData/LocalAppData folder.” Whitelisting is one of the technologies described by ENISA [5] as a good practice that is often not implemented today.
- ** User authentication and access rights:** A study by KPMG [32] showed that among the most important data security vulnerabilities in healthcare are breaches or data theft by employees. This indicates that managing access rights is an essential requirement. The first part of this is the user authentication. ENISA [21] defines strong authentication as a baseline security element for ICT products in healthcare, which “shall provide and support strong authentication mechanisms for all accounts. If authentication is unsuccessful the product shall not allow any user specific activities to be performed.” They furthermore state [5] that “it is essential that authentication is a strong and non-reputable, and that privileges are fine-grained.” NTT security [3] recommends organizations to “follow the principle of least privilege for file access on servers and other systems available through file shares. This reduces the impact of ransomware encrypting files on these systems.” They also recommend to “limit administrator-level privileges as much as possible. Require people to use administrator accounts only when necessary and to use regular user accounts for all other tasks. This reduces the chances attackers will be able to gain immediate access to administrator privileges through a single attack.” The recommendation to assign access rights based on the principle of least privilege can be found in many guidelines [2, 5, 21, 25]. Historically, this is certainly not a strong point of the PACS community where until 2004 the DICOM standard did not even permit a diagnostic workstation to notify the PACS server about the user identity when queries were performed or images were accessed. The importance of this topic has increased, however, with the advent of “enterprise PACS.” Disciplines such as pediatrics, surgery and dermatology store sensitive images, e.g., of children or plastic surgery, unlimited access to which across the complete enterprise may not be acceptable. Furthermore, hospitals nowadays often deploy so-called vendor neutral archives (VNA) where multiple systems store their data on common storage servers (storage area network or network attached storage, SAN/NAS). The potential of a single system that gets infected with malware and has unlimited write access to damage not only the PACS archive, but disrupt the operation of multiple IT systems makes the management of access rights an important requirement.
- ** Regular updates and patches:** ENISA [5] explains that “regular patching and updating of software is essential to avoid the exploitation of known vulnerabilities as well as to ensure the detection of attacks using known paths. Accordingly, in smart hospitals, patches and updates are not only important for networked medical devices and clinical networked information systems, for example, but also for firewalls, antivirus software and other software-based security measures.” Sittig et al. [25] recommend that “personnel in the organization responsible for maintaining all of the computers’ operating systems, application software, browsers and plug-ins, firmware, and anti-virus software should ensure that they are up-to-date with the latest patches. Before applying any patches, health IT professionals should thoroughly test them.” NTT Security [27] recommends organizations to “prioritize patching efforts based on […] exposure, most critical systems, and highest risk vulnerabilities.” The US Department of Justice [2] recommends to “consider using a centralized patch management system.”
- ** Virus and malware protection:** ENISA [5] recommends that “computers should run antimalware and anti-spam software (also known as antivirus) to detect and remove or quarantine malicious software. This includes but not limited: medical devices, IT equipment, health information systems, SCADA [Supervisory Control and Data Acquisition] and Cloud-based data and application services, etc.” NEMA [18] adds that “virus protection mechanisms are good practice to combat (known) threats, and suppliers should ensure that virus definition and updated virus protection patterns do not affect clinical/operational functionality by conducting basic assurance testing of the imaging device.” The US Department of Justice [2] recommends to “set anti-virus and anti-malware programs to conduct regular scans automatically.” Finally, Sittig et al. [25] suggest that “organizations should consider blocking email messages with potentially weaponized attachments” (i.e., file types that may contain executable code).
- ** Encryption:** In cybersecurity, usually three states of data are distinguished: “in use,” “at rest,” and “in transit.” Data is in use when currently read or written by some application. Data is at rest when it is stored but not currently used. Data is in transit when it is being transmitted, either using a network connection or a storage medium such as a compact disk (CD) or a flash memory stick. While it is obvious that data cannot be fully encrypted while being used (it must be in clear-text form at least in the system’s memory), data can be encrypted while being “at rest” or “in transit.” ENISA [5] states that “encryption is one of the most common solutions used in hospitals, mainly because of the criticality and sensitivity of the data at rest, in transit and in use. Health information data stored in third party providers, as well as the ones stored in the hospitals should be encrypted.” Furthermore, they recommend [20] that organizations “ensure a proper and effective use of cryptography to protect the confidentiality, authenticity and/or integrity of data and information (including control messages), in transit and in rest. Ensure the proper selection of standard and strong encryption algorithms and strong keys, and disable insecure protocols. Verify the robustness of the implementation.” Furthermore they recommend [20] to “ensure that communication security is provided using state-of-the-art, standardized security protocols, such as TLS (Transport Layer Security) for encryption.” NEMA confirms that encryption of data “in transit” not only affects wide area transmission of data [18]: “Secure communication is essential when transmitting Protected Health Information (PHI) and associated information between devices and recipients, whether internal to the organization or with external parties.”
- ** Audit trail/logging:** The purpose of an audit trail (or audit log) is to keep a permanent record of all events related to the creation, modification, use and transmission of protected health information. NEMA [18] suggests the introduction of “audit logs for imaging equipment and imaging informatics systems.” ENISA [20] proposes to “implement a logging system that records events relating to user authentication, management of accounts and access rights, modifications to security rules, and the functioning of the system. Logs must be preserved on durable storage and retrievable via authenticated connections.” A standardized message format and communication protocol for audits related to PACS and medical imaging is described in the Integrating the Healthcare Enterprise (IHE) “Audit Trail and Node Authentication” (ATNA) integration profile, which is part of the IHE IT-Infrastructure Technical Framework [33], and in the related DICOM Audit Trail Message Format Profile [34]. It should be noted, however, that an audit trail server is a very attractive cybersecurity target in itself and must, therefore, be appropriately protected.
- ** Network monitoring and intrusion detection:** Sittig et al. [25] recommend that “all organizations should develop a network and user activity monitoring system that conducts surveillance for suspicious activities such as receipt of email messages from known fraudulent sources, executable email attachments, unexpected changes in key files on network-attached drives, unknown processes encrypting files, or significant increases in network traffic on unexpected ports.” The purpose of this monitoring is “to detect suspicious activities and identify and address security problems before they cause harm.” ENISA [5] also recommends the implementation of monitoring and intrusion detection systems, which are “are solutions that monitor a network or systems for malicious activity or policy violations. Violations that are detected are typically reported directly to a member of the IT staff or collected in a central database for further analysis […].” It should be noted that intrusion detection is a field of active research, since such systems need to react on certain “patterns” of system behavior. For example, Maimó et al. [35] describe a machine-learning (i.e., “artificial intelligence”) approach that can identify ransomware attacks with high accuracy, thus improving response times and limiting damage.
- ** Protection of mobile devices:** The rise of mobile devices such as smartphones and tablet computer has given rise to the concept of “Bring Your Own Device” (BYOD), where employees use personally owned mobile devices at work, e.g., to read e-mail, receive notifications, or access medical images from remote. This creates a new attack vector since these devices might get compromised by malware, or get stolen. Liu et al. [22] report that most breaches of health data “occurred via electronic media, frequently involving laptop computers or portable electronic devices.” ENISA [5] states that the “lack of a clear and strict BYOD policy can be great vulnerability,” and that “hospitals should typically prevent patients/employees from connecting their own personal devices to hospital systems (including via Wi-Fi, Ethernet, or VPN (virtual private network)), and where this is not appropriate apply effective technical controls to protect the hospital and the network infrastructure from rogue or compromised devices.” They recommend hospitals to “create a BYOD and mobile device policy for users; as this is a component of a smart hospital ecosystem this needs to become a priority.” In detail, they recommend the introduction of mobile device management (MDM) solutions, “a particular type of asset and configuration management systems that allow changing configurations and working with logs. They allow better protecting the sensitive data that may be stored on mobile devices. Logs of system events sometimes allow detecting malicious actions or system failures.” Furthermore, they note that the installation of antivirus software could “also be a prerequisite for remote care equipment and users mobile devices (in BYOD) to connect to the hospital systems.” The lack of a BYOD policy or control of that policy is noted by ENISA [5] as one of the good practices that is often not implemented today.
  As described above, the ENISA study [5] identified a number of gaps, i.e., good practices that are often not implemented in hospitals. The following gaps are related to technical mitigation measures, in addition to the two practices already discussed in this section:
- ** Automated asset inventory discovery tools:** These are tools for maintaining an inventory of hardware and deployed software. The tools provide a “discovery” feature to either scan the network for active devices, or to identify devices by passively analyzing their network traffic. ENISA [5] writes: “Hospitals adopting IoT components need to monitor how these sensors interact with medical devices and systems, and if information collection process is always correct. To achieve this, an automated asset inventory discovery tool is needed. This tool enables systems managers to track of all assets and being able to use different discovery methods in case of a disruption. Lack of this makes smart healthcare systems more vulnerable to availability and integrity attacks.”
- ** Ensuring secure configurations:** ENISA [5] states that “hospital information security managers should include cyber security in the requirements when purchasing new equipment when building their smart hospital. Security should be built-in but also (due to the great number of legacy systems) integratable; patching and updating should be a regular task of information security officers.” One aspect of ensuring secure configurations is that security mechanisms and algorithms that are considered secure today may be discovered to be faulty in the future, or may become insecure simply because of the general increase in computer speed and memory available to attackers. For systems with a long lifetime, such as medical imaging devices, this means that the security features may need to change over time. ENISA therefore demands [21] that “the provider shall guarantee support throughout the agreed lifetime of the product such that the system can work as agreed and is secure.”
- ** Client certificates:** According to ENISA [5], these certificates are needed “to validate and authenticate systems: Authentication and authorization is significant in the context of smart hospitals; however due to the disperse nature of its components this is not a priority.”
- ** Remote administration over secure channels:** ENISA [5] states that “Remote services are a benefit of smart hospitals. Introducing this new function in a traditional hospital requires more than a regular monitoring system. The remote devices need to be monitored and sometimes even controlled through a central system over secure channel.” The fact that remote services are a viable vector for cyberattacks is illustrated by a news report by ProRepublica published in September 2019 [36] that details such an incident and concludes that the attack “illustrates a new and worrisome frontier in ransomware—the targeting of managed service providers, or MSPs, to which local governments, medical clinics, and other small- and medium-sized businesses outsource their IT needs.”

#### Organizational Mitigation Measures

A study by IBM [37] reports: “What is fascinating—and disheartening—is that over 95 percent of all incidents investigated recognize ‘human error’ as a contributing factor. The most commonly recorded form of human errors include system misconfiguration, poor patch management, use of default user names and passwords or easy-to-guess passwords, lost laptops or mobile devices, and disclosure of regulated information via use of an incorrect email address. The most prevalent contributing human error? ‘Double clicking’ on an infected attachment or unsafe URL [Uniform Resource Locator].” This makes clear that security awareness training for users and other organizational mitigation measures play an important role in implementing cybersecurity as the third mainstay, together with physical and technical mitigation measures. The following recommendations can be found in literature:

- ** User training and simulation:** Many studies point out the importance of raising awareness and training the users of IT systems related to cybersecurity. ENISA [5] defines the difference between awareness-raising and training activities as follows: “while awareness-raising activities target rather broad audiences and are intended to make individuals recognise security risks and respond appropriately, training is more formal and has the goal of building knowledge and skills. With respect to training needs in smart hospitals, creating an understanding of the central systems and their components as well as the interactions among systems and components is of particular importance.” NTT Security [3] recommends organizations to “require regular security awareness training for all users so they are up to speed on phishing, social engineering, and ransomware, especially how to identify attacks, what to do if they need help, and how to report possible attacks.” Argaw et al. [12] advise hospitals “to develop training programs that are at least annually re-evaluated and amended based on recent events. Training is recommended in privacy policies, data leakage prevention, and workplace social media use, but is especially stressed in digital hygiene—good practices of digital security such as choosing strict privacy settings and strong password protection.” Sittig et al. [25] furthermore suggest that “in addition to making end-users aware about the risks and proper responses to fraudulent email messages with attachments, health IT professionals should conduct simulated phishing attacks by sending fake (but safe) email messages or links to websites that appear to be from legitimate sources,” a recommendation that is also put forward by NTT Security [3]. Argaw et al. [12] add that “hospitals should run IT security drills and mock system recovery exercises in order to keep all members vigilant.” ENISA [5] describes the “lack of training and awareness-raising programs” as one their identified gaps, i.e., a good practice that is often not implemented today in hospitals.
- ** Penetration testing:** This term refers to the performance of authorized simulated cyberattacks on a computer system by IT security experts in order to evaluate the security of the system. NEMA [18] recommends that device suppliers should “pre-test the penetrability of a device in its intended operational environment to determine and document constraints operators must consider in the field.” ENISA [20] recommends that hospitals should “conduct periodic audits and reviews of security controls to ensure that the controls are effective. Perform penetration tests at least biannually.” The US Department of Justice [2] even recommends organizations to “conduct an annual penetration test and vulnerability assessment.”
- ** Incident management:** ENISA [6] reports that “according to the findings of the conducted surveys and interviews, one of the top priorities in security appears to be the management of incidents. Many countries pointed out that incident reporting is the key for improving security planning and measures.” Sittig et al. [25] recommends that, “following any unexpected extended system downtimes, whether caused by ransomware or some other human or naturally occurring event, the organization should convene a multi-disciplinary investigation team consisting of key administrative and clinical stakeholders and Health IT professionals to review the event and its management, identify potential root causes, and discuss future prevention or mitigating procedures.”

### Cybersecurity in PACS and Medical Imaging

While there is a large number of publications discussing the cybersecurity requirements of information technology in healthcare in general, the number of publications that specifically discusses security requirements of PACS and medical imaging is much smaller, and many of the publications available focus on the security requirements for the exchange of medical images over public networks, e.g., in the context of teleradiology/telemedicine applications or Electronic Health Records (EHR). Strickland [38] discusses the risks associated with the deployment and operation of filmless PACS, although not focused on cybersecurity. Desjardins et al. [39] provide an overview of cybersecurity issues related to DICOM and derive recommendations for radiologists, IT staff, and standards bodies. Ruotsalainen [40] discusses requirements for trustworthy teleradiology models, focusing on organizational aspects. These requirements include “a common security policy that covers all partners and entities, common security and privacy protection principles and requirements, controlled contracts between partners, and the use of security controls and tools that supporting the common security policy. The security and privacy protection of any teleradiology system must be planned in advance, and the necessary security and privacy enhancing tools should be selected […] based on the risk analysis and requirements set by the legislation.” Gutiérrez-Martínez et al. [41] discuss how to implement an information security management system for a large-scale PACS in accordance with ISO/IEC 27002:2013 [13]. Finally, a recent NIST publication [42] proposes a reference architecture for a secure PACS using commercially available, standards-based tools and technologies, including segmented network zones, authentication and access control, and a holistic risk management approach. The publications discussed below each present individual “building blocks” for a secure storage or exchange of medical images.

#### Image De-Identification

In many cases, it is legally required that images are de-identified before they are transmitted to a third party or submitted to a database. Typical use cases for image de-identification include clinical studies, research, and teaching databases. However, de-identification can also be useful for teleradiology applications when possible because irreversible anonymization significantly reduces the security requirements for image sharing. In the case of the DICOM standard, image de-identification is not trivial though. In its current edition [34], the DICOM standard defines more than 4500 attributes (fields) that may be present in the “header” of a DICOM image, and for each attribute it must be decided whether or not it may contain confidential information that must be removed, and if so, how the information can be removed without negatively affecting the correctness and consistency of the image or the study to which the image belongs. This includes many free-text comment fields that may or may not contain confidential information. Furthermore, DICOM allows vendors to add vendor-specific “private” attributes to a DICOM image that may also contain patient identifying information. Finally, there are cases where patient identifying information is present in the image bitmap itself (e.g., most ultrasound devices render the patient name into the image data) or where the patient’s face could be reconstructed from an image set (e.g., head CT).

The DICOM standard itself provides detailed instructions for the de-identification of images and associated data in the form of the “Basic Application Level Confidentiality Profile,” which was added to the standard in 2011. It provides a list of some 350 DICOM attributes that need to be taken into account when de-identifying, and provides instructions on how to handle these. It furthermore offers 10 different options (e.g., “clean pixel data,” “retain longitudinal temporal information,” and “retain device identity”) that allow users to adapt the de-identification process to the requirements of their use case.

Freymann et al. [43] describe the de-identification requirements for research databases under the US Health Insurance Portability and Accountability Act (HIPAA) legislation and present an implementation of the DICOM Basic Application Level Confidentiality Profile for the National Biomedical Imaging Archive Project. Robinson [44] provides an overview of literature and available tools for de-identification of DICOM images and discusses cases where patient identifying information may be present in the image pixel data, such as Ultrasound and Nuclear Medicine images, screen captures, scanned documents, post-processed images and the output of some computer-aided detection systems. Clunie et al. [45] discuss the special case where patient identifying information is embedded in the image pixel data of images that have been subjected to an irreversible (lossy) image compression using the JPEG (Joint Photographic Expert Group) compression algorithm, something that is very common for example with Ultrasound cine-loops. In this situation the sequence of decompression, de-identification and re-compression would lead to a decrease in image quality, which is undesirable. They describe how a property of the JPEG algorithm, which compresses small blocks of the image independently, enables a de-identification tool to only modify those parts of the image containing patient identifying information, without changing the other parts. Finally, Aryanto et al. [46] discuss how a de-identification process could be integrated into an image sharing network based on the IHE Cross-Enterprise Document Sharing for Imaging (XDS-I) integration profile.

#### Transport Security

The DICOM network protocol, which is commonly used to exchange medical images, is based on TCP/IP (Transmission Control Protocol/Internet Protocol). The Internet Engineering Task Force (IETF), the governing body of the Internet protocol, has defined a number of standards and specifications adding security features to the otherwise largely unprotected TCP/IP. In particular, TLS [47] is a TCP/IP-based network protocol that enables “applications to communicate over the Internet in a way that is designed to prevent eavesdropping, tampering, and message forgery.” TLS can be applied to all network protocols based on TCP, such as DICOM, HL7, or Webservices such as the ones used for IHE XDS-I. Originally defined in 1999 as TLS 1.0, the standard is continuously updated in order to remain secure, implementing results from cryptographic research and taking into account the increasing power of computers. The DICOM standard offers a set of “secure transport connection profiles” that describe how to use TLS with DICOM network connections. The first of these, the “Basic TLS Secure Transport Connection Profile” was added to the standard in early 2000, and an open source reference implementation of this new DICOM extension was publicly demonstrated at the Radiological Society of North America’s (RSNA) Annual Meeting in the same year [48]. For systems that do not support TLS, a gateway can be implemented that accepts “normal” DICOM network connections and forwards these using TLS. An early implementation of such a gateway was already described by Thiel et al. [49] in 1999. Alternative approaches to transport security are the use of VPNs, which are discussed by Nyeem et al. [50], and encrypted e-mail transfer, as described by Schütze et al. [51].

#### Selective Encryption of the DICOM Header

In some cases, it is desirable to selectively encrypt only those attributes of the DICOM header that contain patient identifying information instead of applying a transport security scheme as discussed in the previous section. Essentially this approach is the same one as used in image de-identification (see the “Image De-identification” section), except that the values of the header fields that are modified during the de-identification process are stored in an encrypted “container” and can be used later to reconstruct the original, fully identified image.

This concept was originally proposed by Thiel et al. [52] of the Medbild project, who implemented teleradiology on an experimental metropolitan high-speed network and noticed that the computers at the time were unable to fully utilize the available network bandwidth when transport encryption was enabled, i.e., encryption slowed down the data transfer significantly. For the majority of imaging modalities, there is no risk of patient re-identification of the image pixel data, which typically makes up more than 99% of the size of a DICOM image. A selective encryption of the header only, therefore, enabled the project to transmit images at “full speed” without sacrificing confidentiality.

This approach was standardized in DICOM as part of the “Application Level Confidentiality Profiles” discussed in the “Image De-identification” section. The attributes and attribute values that are modified during the de-identification process are copied into a separate DICOM dataset, which is encrypted using a format called Cryptographic Message Syntax (CMS) [53] and then stored in the header of the de-identified DICOM image in encrypted form. Depending on the type of key management chosen, only the owner of the private recipient key or anyone in possession of the pre-shared encryption key can reconstruct the original image.

#### Full Encryption of DICOM Images

An alternative to either a transient transport encryption or a selective encryption of DICOM header fields is obviously the full encryption of DICOM images including both header and image pixel data. A standardized solution for this approach is offered by the DICOM standard, which since 2001 defines the “Basic DICOM Media Security Profile,” primarily intended for the secure exchange of medical images over storage media. This profile defines an encrypted DICOM file format where the complete DICOM image is encapsulated using CMS [53], the same format that is also used for selective encryption. Encryption is either based on a password that must be known to the recipient of the storage medium, or based on a Public Key Infrastructure (PKI). In this case, a certificate containing the recipient’s public key is used during the encryption process, and the recipient must own the corresponding private (secret) key in order to decrypt the file. Encrypted files can optionally contain a digital signature as part of the CMS envelope. The encrypted DICOM file format has one significant drawback: it cannot be used for DICOM network transmissions, i. e. image files must be decrypted before they can be forwarded over a network connection.

Several alternative approaches to the encryption of DICOM images have been proposed in literature. Al-Haj [54] proposes an approach that combines full encryption of the pixel data with selective encryption of header data and includes a digital signature. Praveenkumar et al. [55] propose the combination of three encryption algorithms for encrypting pixel data, a Latin Square Image Cipher, a Discrete Gould Transform and Rubik’s Encryption. Natsheh et al [56] propose a specific encryption scheme for multi-frame images that uses the first frame as a “one time pad” for the following frames and only encrypts the first frame using a conventional encryption algorithm. All these alternative approaches suffer from the fact that they are non-standard and that only very limited cryptanalysis has been applied to them. While standards-based approaches such as TLS or CMS have been studied by many cryptography experts over many years, only very limited knowledge is available about possible weaknesses in the alternative approaches.

#### Digital Signatures

The need to guarantee the integrity and authenticity of medical images has been recognized early. Wong et al. [57] proposed the use of digital signatures and timestamps to prevent an unauthorized modification of images already in 1995. The DICOM standard introduced the concept of digital signatures and trusted timestamps in 2001 with the addition of the first Digital Signature Profiles. These profiles enable one or more digital signatures to be applied to a complete DICOM image or parts thereof, and then to be embedded in the DICOM header, which makes sure that the digital signature is always stored and transmitted as part of the signed image or document, together with the certificate of the signer, which permits a validation of the signature by any system that receives the image. An early implementation of DICOM digital signatures is described by Riesmeier et al. [48].

Digital signatures are based on public key cryptography. Since the cryptanalysis of the algorithms used might advance in the future and since computers are getting faster over time, an algorithm that is considered secure today might be considered insecure in the future. For this reason, digital signatures have a finite lifespan—typically a few years. While there is no universal model that defines at which point in time a signature loses its trustworthiness, the expiry of the certificate identifying the signer or the revocation of a certificate (e.g., in the case of key theft) are possible end points. For digital signatures that need to remain valid over a long time, three possible solutions are discussed in literature:

- A new digital signature can be affixed to each signed document (image) before the old signature expires. While theoretically possible, this approach that is not practical with large archives of image data.
- Certified timestamps can be used. In this approach, the digital “fingerprint” (cryptographic checksum) of the image, which is the basis for the digital signature, is transmitted to a trusted third party operating a timestamping service. The trusted third party adds date and time information to the “fingerprint” and signs both timestamp and fingerprint as a proof that the fingerprint has been received at a certain date and time. Since the signatures on the timestamps also expire, a new certified timestamp of the old certified timestamp may be required a few years later. The DICOM standard permits the use of such certified timestamps, but does not require them.
- The digital “fingerprint” of the image can be published in a blockchain, which is designed as a distributed ledger that makes retrospective modifications practically impossible. Since transactions in a blockchain also carry a timestamp, the blockchain implements the role of a certified timestamp without the need for a trusted third party. A further overview of this topic is provided by Shuaib et al. [58].

Finally, there is the practical problem that most imaging modalities simply do not support digital signatures. Kroll et al. [59] proposed the use of an embedded system (i.e., a very small computer) as a gateway that receives the images from one modality, adds a digital signature to each image, and then forwards the images to the image archive.

#### Watermarking

A relatively large number of publications discusses the concept of “digital watermarking” of medical images. Watermarking describes a process whereby information (such as identity information or a digital signature) is “hidden” in the image pixel data in the form of high frequency information (“snow”) that is largely invisible to the human eye but recognizable by an algorithm that specifically checks the presence of a digital watermark. In general, watermarks are used for three main purposes: authentication, integrity control and the hiding of information in the image (steganography). Watermarks intended for authentication are usually designed as “robust” watermarks that remain decodable even if certain modifications such as a lossy compression have been applied to the image. Watermarks for integrity control are similar in concept to a digital signature: If the image is modified in any way, the watermark should become invalid. This concept is referred to as “fragile” watermarks. The concept of “data hiding” has been used in teleradiology applications where de-identified images are stored and transmitted without encryption, and the patient identifying information is “hidden” in the image data as a watermark. A good overview of digital watermarking and requirements for use in medical imaging is presented by Nyeem et al. [50]. Singh et al. [60] also provide a comprehensive overview of the topic.

When used for authentication purposes, the main advantage of digital watermarks over digital signatures is that watermarks are difficult to remove from the image, whereas digital signatures can easily be removed. In an environment where both digitally signed and unsigned images are in use, an attacker might simply choose to remove the signature from an image that has been tampered with, thus avoiding detection. This is much more difficult when watermarking has been applied. The main disadvantage of digital watermarks is that they cause a degradation of image quality, which is most often not acceptable for medical images. Therefore, most publications on this topic propose that the “region of interest” of the image is identified prior to the application of the watermark, which is then only hidden in the remaining parts of the image, i.e., the “background.” Unfortunately, the identification of the region of interest cannot be fully automated for all types of images. Furthermore, with the increasing importance of artificial intelligence applications in medical imaging, the effect of digital watermarks on the training and use of convolutional neural networks is an issue of concern: It is possible that a training dataset containing images with and without watermarks causes the neural network to “learn” to detect the presence or absence of the watermark instead of some clinical feature visible in the images, thus negatively affecting the accuracy of the image classification.

The main problem with watermarking is, however, that is has never been standardized, so all approaches are proprietary. Furthermore, watermarking typically depends on some shared secret (e.g., a secret key) that the recipient must know in order to identify the watermark. Without such a shared secret, a malicious attacker could simply check for the presence of a watermark and modify the image until the watermark is not detectable anymore, which is essentially the same as removing a digital signature. When a shared secret is used, however, the question is how this secret can be shared between sender and receiver such that an attacker can neither read nor remove it.

#### DICOM File Preamble Cleansing

The DICOM file format, which is used when exchanging DICOM images on storage media or using DICOM web services, specifies that the first 128 bytes of the file, the so-called preamble, may contain arbitrary information not used by a DICOM reader. In 2019, Ortiz published a report and a proof of concept [61] that shows that the preamble can be abused to construct files that are at the same time a valid DICOM image and a valid Windows “Portable Executable” program. This issue was registered in the National Vulnerability Database of the National Institute of Standards and Technology (NIST) as CVE-2019-11687 and rated with a severity base score of “HIGH.” The Common Vulnerabilities and Exposures (CVE) description [62] explains that “to exploit this vulnerability, someone must execute a maliciously crafted file that is encoded in the DICOM Part 10 File Format. PE/DICOM files are executable even with the .dcm file extension. Anti-malware configurations at healthcare facilities often ignore medical imagery.” As a press release of the DICOM committee [63] explains, “a user might be convinced to execute the file via social engineering. Alternatively, a separate malicious actor that knew about the embedded executable and had access to the modified file could install and execute the malware. This type of intrusion is referred to as a multi-phase attack.” A “frequently asked questions” document accompanying the press release [64] recommends that applications that read DICOM images from media or receive them using DICOMweb should “ensure that if a preamble exists, it is a known preamble such as TIFF. If not, the preamble should be cleared (set to 00H).”

It should be noted that the DICOM file preamble is not transmitted when an image is sent over a network using the DICOM network protocol, e.g., between PACS and viewing workstation. That means that a network transmission of a DICOM image automatically “cleans” the image by removing the header that marks the image as executable code. In other cases, applications that handle DICOM files should perform a cleansing of the file preamble.

## Discussion

When comparing the two bodies of literature presented in the “Review” section, there is a remarkable disconnect: While literature on general cybersecurity and healthcare IT cybersecurity often tries to present comprehensive, multi-layered security approaches (“defense in depth”), the available literature on cybersecurity for PACS and medical imaging focuses on individual aspects such as the de-identification of images, the protection of images against undetected modification, or the encryption of images in transit. In general, many publications related to teleradiology and medical image sharing seem to imply that the local network within a hospital is secure, and cybersecurity considerations only become necessary once images are transmitted beyond the perimeter of the hospital network. While this may have been a sensible approach 20 years ago, it certainly is not sufficient anymore today. A multi-layered security strategy for PACS would mean, for example, that

- The file preamble is cleansed when receiving or importing DICOM files, but nevertheless antivirus software and application whitelisting are used on the DICOM viewers receiving the files.
- The integrity of images is secured with digital signatures, but nevertheless the network transmission of the images uses public-key certificates to authenticate authorized image sources.
- Images are de-identified when possible, but nevertheless the encryption is used for the transmission.
- Network communication in the PACS network is migrated to encrypted and authenticated transmission, but nevertheless the network is secured with firewalls and network segmentation.
- The PACS network is secured with firewalls and network segmentation, but nevertheless an intrusion detection system is deployed to detect breaches of the network security.
- Access to images in the PACS is controlled by user specific access rights, but nevertheless an audit trail is deployed that allows to analyze, “who did what.”
- Antivirus software and whitelisting are deployed where possible in order to prevent ransomware attacks, but nevertheless a secure, remote backup of the database is maintained.

In this approach, a breach of a single mitigation measure would not jeopardize the security of the PACS network. Articles that discuss concrete attack scenarios against PACS networks and mitigation measures have recently been published by Desjardins et al. [39] and Eichelberg et al. [EKK20]. Furthermore, the NIST Cybersecurity Practice Guide on Securing PACS [42] may arguably be the first attempt to combine all building blocks into an exemplary comprehensive solution. Certainly, further work in this direction will be needed in the future. The good news is that hospitals are not helpless with regard to cybersecurity: the building blocks for a multi-layered security strategy in PACS networks exist—at least conceptually—and many of them can be readily deployed. While such solutions need to be maintained and updated continuously, and while human error will always be a factor that needs to be taken into account, a multi-layered security strategy will make it much harder for malicious actors to cause damage or disruption, and reduce the impact of malicious attacks even if a single phase of attack is successful. Therefore, cybersecurity is more an issue of funding, implementation and maintenance than a fundamental feasibility issue. In the past, cybersecurity may have been underrated and underfunded in many hospitals, but the increasing number of widely publicized security incidents such as the ones discussed in the introduction will certainly help in changing this, by drawing attention to the importance of cybersecurity. Today, the PACS and medical imaging specific measures presented in the “Cybersecurity in PACS and Medical Imaging” section may be implemented in teleradiology settings, but they are not commonly used for routine operation within the hospital network, which would provide an important contribution to a multi-layered “defense in depth” for the imaging network. In the view of the authors, the following factors contribute to this situation:

- Lack of device support: Very few imaging modalities support encryption or can apply digital signatures to medical images. This is largely a “chicken and egg” problem: users have little incentive to demand support for these technologies when procuring devices as long as most of their other devices do not support them, and there is little incentive for the vendors to implement and offer these technologies as long as there is little customer demand. While encryption in the form of transport security can be added to an operational PACS network by means of gateway hardware or software, and while encryption of data “at rest” (e.g., in the PACS or VNA archive) can be implemented in individual products, digital signatures require support both in the devices creating images or documents, and the devices reading and processing or displaying these images or documents.
- Lack of a manageable public key infrastructure: A deployment of transport security (i.e., network encryption), selective or full encryption of DICOM images or digital signatures requires the management of certificates and private keys on which all of these protocols rely. A certificate is digital file that contains a public key, identity information about the owner of that public key (e.g., person or system name), a period of validity, and a digital signature by a trusted “certification authority” (CA) that has verified the identity of the owner. Each certificate is associated with a “private key,” which is never transmitted and must be kept secret. All systems that initiate or accept encrypted connections, encrypt or decrypt files, or create or verify digital signatures must be provided with a pair of certificate and private key, and with rules describing which other certificates should be considered trustworthy (this is usually either an explicit list of all trusted certificates, or a list of CA certificates, in which case all certificates issue by one of these CAs would be considered trustworthy). Since all certificates have a limited validity, they must be updated regularly and automatically. Surprisingly, no well-accepted standard for this task exists today that would be suitable for a PACS environment.

The lack of device support will certainly change once hospitals start to request security features as part of their procurement process for PACS and imaging devices. The lack of suitable solutions for a PACS public key infrastructure suggests that standardization work will be required first, for example in the form of an IHE integration profile.

## Summary

Table 1 summarizes the cybersecurity measures applicable to PACS and medical imaging that are proposed in literature and have been discussed in this article. The “user/vendor” column indicates which of these measures can be implemented by the user (e.g., hospital) directly, and which ones require support from the device vendors in addition to the user’s effort. The “CIA triad” column indicates to which of the three information security goals of confidentiality, integrity, and availability each measure contributes. Mitigation measures that only indirectly contribute to the CIA triad, such as user training, are shown in parentheses. Finally, the “references” column lists references to sources that discuss each measure further.

**Table 1:** Summary of cybersecurity mitigation measures proposed in literature. CIA, confidentiality, integrity, availability

**Mitigation measure**	**User/vendor**	**CIA triad**	**References**
**Physical mitigation measures (**“Physical Mitigation Measures” **section)**			
Keep file servers in secure areas safeguarded from unauthorized access and environmental threats	U	(CIA)	[13, 14, 23]
install security cameras in server rooms	U	(CIA)	[24]
**Technical mitigation measures (**“Technical Mitigation Measures” **section)**			
Perform regular backups	U/V	A	[2, 13, 23, 25]
Use firewalls and network segmentation to prevent network intrusion	U	(CIA)	[5, 13, 23, 26, 29–31]
Disable unused physical network and USB ports	U	(CIA)	[20, 25]
Use whitelisting for permitted applications	U/V	(CIA)	[18, 25]
Implement user authentication and define and enforce access rights	U/V	C	[13, 14, 23, 32, 66]
Install updates and patches on a regular basis	U/V	(CIA)	[5, 13, 25]
Install antivirus software	U/V	(CIA)	[5, 18, 23, 25]
Use encrypted network transmissions	U/(V)	CI	[5, 18, 20]
Use encrypted document storage	U/V	CI	[5, 18, 20]
Deploy an audit trail	U/V	(CI)	[13, 14, 20, 33, 34]
Deploy network monitoring and intrusion detection tools	U	(CIA)	[5, 25, 35]
Define and enforce a mobile device policy	U	(CIA)	[5, 13, 14, 23]
Deploy automated asset inventory discovery tools	U	(CIA)	[5]
Ensure that system configurations are updated to remain secure over time	U/V	(CIA)	[5, 21]
Deploy a public key infrastructure providing client certificates	U/V	CI	[5, 65]
Enforce remote administration to be performed over secure channels	U	C	[5, 36]
**Organizational mitigation measures (** “Organizational Mitigation Measures” **section)**			
Perform regular user training and simulate cybersecurity incidents	U	(CIA)	[5, 12, 13, 25]
Perform regular penetration testing	U	(CIA)	[20]
Define and implement incident management procedures	U	(CIA)	[6, 13, 25]
**Medical imaging specific mitigation measures (** “Cybersecurity in PACS and Medical Imaging” **section)**			
Use de-identified images where possible	U/V	C	[34, 43–46]
Implement DICOM transport security or selective encryption of DICOM headers	U/(V)	C	[48–52]
Store DICOM files in encrypted format	U/V	C	[34, 53]
Use digital signatures or watermarking techniques to protect image integrity	U/V	I	[48, 50, 57–60]
Cleanse file preamble when handling DICOM files	U/V	(CIA)	[61, 62, 64]

In some cases, vendor support is needed for the installation of additional software (such as backup or antivirus software), or the operating system configuration (for the use of whitelisting features), which in the case of medical devices may require consideration in the certification process (e.g., risk management). The installation of updates and patches and the maintenance of secure system configuration over time will require the provision of validated updates by the vendor. Other measures will require vendor support in the product’s software: user authentication and access rights, the creation and verification of digital signatures or watermarks, encrypted document storage, submission of audit records to a central audit trail server, and the use of de-identification or selective encryption techniques where possible can only be implemented by the product vendor. Finally, the deployment of public key infrastructure that provides for an automated distribution and renewal of client certificates requires a collaboration between the user organization (which is responsible for the PKI) and the vendor, which must implement support for the PKI in the product. As discussed above, transport security may be implemented by means of gateways or directly in the product, so this could be implemented with or without vendor support.

In summary, the building blocks for a multi-layered security strategy in PACS networks exist today, at least conceptually, and many of them can be readily deployed. While such solutions need to be maintained and updated continuously, and while human error will always be a factor that needs to be taken into account, a multi-layered security strategy will make it much harder for malicious actors to cause damage or disruption, and reduce the impact of malicious attacks even if a single phase of attack is successful, if hospitals decide to assign the resources required. There are, however, some practical issues that hinder implementation. This includes a lack of support for security features in today’s medical imaging products and a lack of practical solutions for managing the public key infrastructure for a PACS and medical imaging network where devices from different vendors, perhaps using different operating systems, need to be continuously provided with client certificates and policies for certificate verification.
